# Recombination Speeds Adaptation by Reducing Competition between Beneficial Mutations in Populations of Escherichia coli

**DOI:** 10.1371/journal.pbio.0050225

**Published:** 2007-08-21

**Authors:** Tim F Cooper

**Affiliations:** School of Biological Sciences, University of Auckland, Auckland, New Zealand; University of Edinburgh, United Kingdom

## Abstract

Identification of the selective forces contributing to the origin and maintenance of sex is a fundamental problem in biology. The Fisher–Muller model proposes that sex is advantageous because it allows beneficial mutations that arise in different lineages to recombine, thereby reducing clonal interference and speeding adaptation. I used the F plasmid to mediate recombination in the bacterium Escherichia coli and measured its effect on adaptation at high and low mutation rates. Recombination increased the rate of adaptation ∼3-fold more in the high mutation rate treatment, where beneficial mutations had to compete for fixation. Sequencing of candidate loci revealed the presence of a beneficial mutation in six high mutation rate lines. In the absence of recombination, this mutation took longer to fix and, over the course of its substitution, conferred a reduced competitive advantage, indicating interference between competing beneficial mutations. Together, these results provide experimental support for the Fisher–Muller model and demonstrate that plasmid-mediated gene transfer can accelerate bacterial adaptation.

## Author Summary

Why have sex? One explanation is that sex is good because it allows beneficial mutations from different lineages to recombine. This reduces competition between mutations in a population and can increase the speed with which the population can adapt to environmental change. This explanation, known as the Fisher–Muller model, has extensive theoretical support; however, it is difficult to test experimentally. Using a simple microbial system I showed that recombination increased the rate of fitness improvement when beneficial mutations were common in the population and had to compete for fixation, but had little effect when mutations occurred rarely. Sequencing of candidate genes revealed the presence of the same beneficial mutation in a number of replicate populations. In the absence of recombination, this mutation took longer to spread and conferred a lower overall competitive advantage, indicating interference between competing beneficial mutations. Together, these results provide direct experimental support for the Fisher–Muller model.

## Abstract 3

Why there is sexual reproduction is an important question. Here the authors show that making bacteria have sex allows faster accumulation and fixation of beneficial mutations.

## Introduction

Understanding the factors that contribute to the origin and maintenance of sex is an important unanswered problem in evolutionary biology [1–4]. A large body of research has developed several theories predicting potential advantages to sex (reviewed in [3]). However, critical support for these theories remains elusive, in large part because of the difficulty of devising suitable tests using traditional comparative approaches. New experimental approaches have begun to address this problem by allowing experiments to be designed to determine the effect of sex in defined environments [5–12].

The Fisher–Muller (FM) model proposes that the advantage of sex results from recombining competing beneficial mutations into one lineage [13,14]. In the absence of recombination, beneficial mutations that occur in the same population, but in different lineages, must compete with one another for fixation. This competition, known as clonal interference, slows the spread of each mutation and can reduce the overall rate of fitness increase [15–18]. Subsequent theoretical analysis of the FM model predicts that recombination can increase the rate of adaptation over a range of population sizes and recombination and mutation rates [19–21].

Despite extensive theoretical support for the FM model, the ability of the model to predict the effect of recombination in biological systems is subject to at least two caveats. First, an advantage of recombination depends on competition between beneficial mutations arising in different lineages being strong enough that the fixation of the higher fitness mutation is appreciably slowed. If that is not the case, competition will not effectively limit the rate of adaptation, and recombination will have little effect [22]. Results of experimental studies have been consistent with the presence of widespread competition between beneficial mutations [18,23–29]. However, the extent to which this competition slows adaptation has not been well-characterised independently of potentially confounding factors such as population size differences between treatments [17,22,30]. Second, the extent and type of interactions occurring between beneficial mutations remains largely unknown. Even in the presence of strong competition between mutations, recombination may not provide a substantial advantage if interactions between mutations cause the advantage conferred by a given mutation to depend on a particular genetic background [31]. This might be the case if beneficial mutations tend to occur as part of co-adapted gene complexes.

An ideal experimental test of the FM model would compare the effect of recombination on the rate of adaptation in treatments that differ solely in the extent of competition between beneficial mutations. In practice this ideal has been hard to achieve. A number of experimental studies have examined aspects of the relationship between recombination and environment [9–12]. These studies have shown an advantage of recombination; however, the treatment regimes also involved differences in environmental [10,12] or life-history [9,11] factors, which can cause differences in adaptive opportunities that are independent of the effect of recombination. One study that minimised these differences found a strong interaction between the effect of recombination and mutation supply but required extensive introgression of genes from non-evolved individuals, complicating interpretation of the mechanism underlying this interaction [11]. Most importantly, none of these studies were able to follow, or even identify, any of the mutational changes underlying adaptation. Without this information it is difficult to determine the relative roles of recombination providing a benefit by (i) bringing beneficial mutations together versus (ii) separating newly arising beneficial mutations from linked deleterious mutations.

To circumvent the problems outlined above and isolate the net effect of recombination across mutation-supply treatments, the study presented here takes advantage of results published by de Visser et al. [18]. In that study, conditions were identified in which the absence of a DNA repair gene, *mutS,* was shown to control the rate of adaptation in evolving populations of the bacterium Escherichia coli by increasing the rate at which new mutations were produced by ∼30-fold. By using the same strains and environmental conditions, I define high and low mutation rate treatments that differ only in the supply of new mutations and thus the degree of competition between beneficial mutations. All other aspects of the environment, including population size, remain constant. To permit recombination I use recombination proficient (rec+) strains made by introducing the F plasmid into the ancestral strains used by de Visser et al. [18]. F mediates recombination by integrating into the bacterial chromosome, where it can facilitate transfer of chromosomal DNA into a recipient cell via conjugation. F is found in many isolates of E. coli and related bacteria, and may play an important role in gene transfer in natural populations [32]. Control recombination deficient (rec−) lines were made by deleting a plasmid gene necessary for transfer.

To test the FM model I evolved eight replicate lines in each of four treatments: rec+, high mutation rate; rec+, low mutation rate; rec−, high mutation rate; and rec−, low mutation rate. All lines were evolved for 1,000 generations in the same constant environment. Following this period of evolution the overall adaptation of each line was estimated. In agreement with the FM model, recombination caused a greater increase in fitness in the high mutation rate lines. Subsequent experiments tracked the dynamics of one of the beneficial mutations underlying this adaptation and found that this increase was associated with a reduction in competition between co-occurring beneficial mutations.

## Results/Discussion

**Figure 1:**
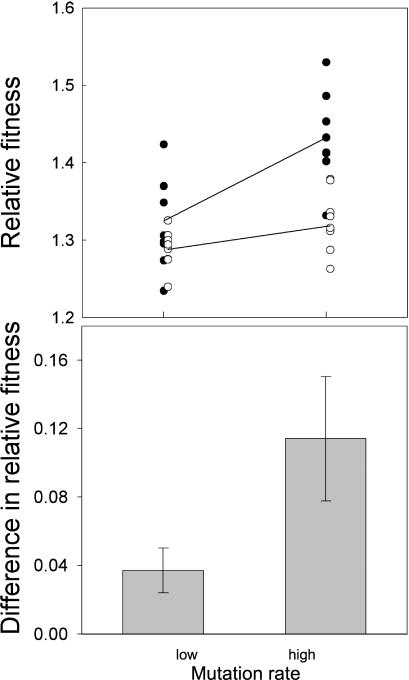
Interaction between Recombination and Mutation Rate on the Rate of Adaptation Top: each point represents the mean of ten replicate fitness estimates for one evolved line. Solid symbols indicate rec+ lines; hollow symbols indicate rec− lines. Lines connect the mean fitness of rec+ and rec− populations evolved in high and low mutation rate treatments. Bottom: differences in relative fitness between rec+ and rec− lines at high and low mutation rate. Error bars are 95% confidence intervals. Recombination caused a significantly greater increase in adaptation in the high mutation rate treatment.

Following 1,000 generations of evolution, fitness increased significantly in all experimental lines; however, the magnitude of this increase varied across treatments. At the high mutation rate, average fitness increased by ∼43% in rec+ lines, and by ∼32% in rec− lines (Figure 1). At the low mutation rate, average fitness rose by ∼32% and ∼29% in rec+ and rec− lines, respectively (Figure 1). A two-way ANOVA found a significant interaction between mutation rate and recombination (*F*_1, 28.72_ = 4.7426, *p* = 0.0378) (Table 1). Therefore, recombination provided a greater advantage at the higher mutation rate.

**Table 1:**
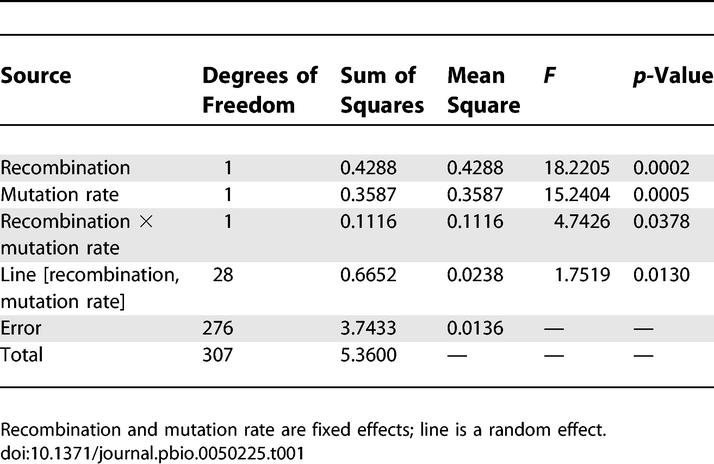
Interaction between Recombination and Mutation Rate

The results presented above are consistent with the FM model, whereby recombination provides an advantage when multiple beneficial mutations compete for fixation by combining them in the same lineage. However, a possible alternative explanation is that recombination was advantageous because it separated beneficial mutations from linked deleterious mutations [10,33–37]. Because the evolving populations were large (*N*_e_ ∼ 1.66 × 10^5^), were evolved for only 1,000 generations, and were started from a single clone, deleterious mutations are extremely unlikely to fix independently of other mutations. However, they can rise in frequency if a beneficial mutation of sufficiently large effect subsequently arises on the same background [34,35]. This linkage is more likely to occur in the high mutation rate treatments, where deleterious and beneficial mutations are more common [34]. Thus, this mutation load model also predicts an increased advantage to recombination in high mutation rate populations. A third possible explanation, the mutational deterministic model, is unlikely to explain my results because the genomic mutation rate in the ancestral strains was substantially lower than that required by the model to produce a benefit to recombination. Previous work has calculated a best estimate of the mutation rate in the low mutation rate ancestral strain as 1.44 × 10^−10^ per basepair per generation [38]. Multiplying this rate by the increase in mutation rate caused by the *mutS* allele and by the genome size gives a genomic mutation rate of only 0.023 (= [1.44 × 10^−10^] × 34.9 × [4.64 × 10^6^ bp])—well short of the genomic mutation rate of ∼1 required by the mutational deterministic model to explain an advantage to recombination. Also, there is no general tendency for deleterious mutations to interact synergistically in this strain, a second prerequisite of the mutational deterministic model [39].

In the light of the alternative mutation load explanation for the observed benefit of recombination, I sought to directly examine the prediction made by the FM model that recombination reduces competition between beneficial mutations. To do this, I compared the dynamics of a focal beneficial mutation as it rose in frequency and ultimately fixed in rec+ and rec− populations. A decrease in the fitness conferred by a focal beneficial mutation, relative to contemporary clones that did not have this mutation, would be consistent with the spread of alternative beneficial mutations in competing lineages.

**Figure 2:**
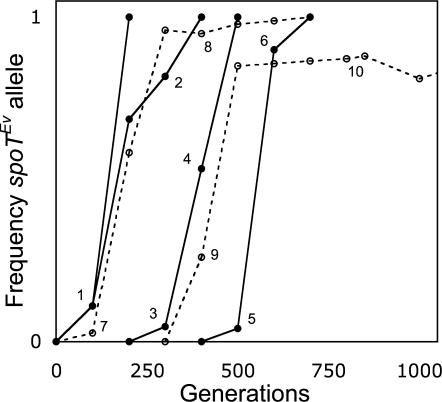
Fixation Dynamics of *spoT* ^Ev^ Beneficial Alleles Solid lines with filled symbols indicate rec+ lines; dashed lines with hollow symbols indicate rec− lines. The *spoT* ^Ev^ allele spread significantly faster in rec+ lines. Numbers indicate sample points from which clones with and without a *spoT* ^Ev^ allele were isolated to allow subsequent fitness measurements (see Figure 3). In one rec− line the *spoT* ^Ev^ allele had not fixed by 1,000 generations. This line was continued for a further 300 generations, after which time the allele had fixed.

A previous study found a mutation in a regulatory gene, *spoT,* that contributed to adaptation of E. coli to an environment nearly identical to the one used here [40]. This gene was thus a candidate for harbouring beneficial mutations in the present study. I sequenced *spoT* in three randomly chosen clones isolated from each of the 16 populations in the high mutation rate treatment and found mutations in four rec+ and two rec− lines. The temporal dynamics of each mutation were examined by screening clones at regular intervals from stored samples of the evolved lines. Figure 2 shows that the dynamics of the evolved *spoT* (*spoT* ^Ev^) alleles were very different in the rec+ and rec− lines. The time between detection and fixation averaged 300 generations in rec+ lines and 900 generations in rec− lines (*t*_4_ = 3.098, one-tailed *p* = 0.018; Mann-Whitney test, one-tailed *p* = 0.066). Thus, a beneficial mutation in the same gene took longer to fix in the absence of recombination.

**Figure 3:**
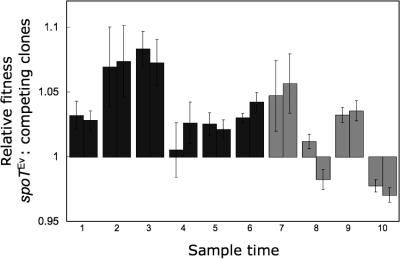
Fitness of Clones Containing a *spoT* ^Ev^ Beneficial Allele in Rec+ and Rec− High Mutation Rate Lines Relative to Five Contemporary Clones Not Containing This Allele The number labels for competing *spoT* ^Ev^ clones follow the sampling numbers indicated in Figure 2. Each bar represents one of two *spoT* ^Ev^ clones isolated at each sample point. dark gray bars indicate rec+ lines; light gray bars indicate rec− lines. Error bars are standard error of the mean.

To identify whether the difference in time taken for *spoT* ^Ev^ alleles to fix in rec+ and rec− lines was due to an increase in competition, the selective advantage conferred by each allele was measured at different time points during its substitution. I isolated two clones that had *spoT* ^Ev^ alleles from three of the rec+ and two rec− lines that fixed a mutation in this gene (the focal beneficial mutation), and estimated their fitness relative to five contemporary clones that were isolated from the same line and time point, but that did not have the focal mutation (Figure 3). It is important to note that these measurements were made relative to clones that did not have the focal *spoT* ^Ev^ beneficial mutation, not against the population as a whole; therefore, a decrease in the relative advantage conferred by the focal mutation indicates an increase in fitness amongst competing clones.

At early time points in their respective selective sweeps (frequency < 0.1 except sample point 9, for which frequency = 0.24), the average advantage conferred by focal *spoT* ^Ev^ alleles, relative to contemporary clones not having this mutation, did not differ significantly between rec+ and rec− lines, being 3.3% and 3.9%, respectively (*F*_1, 3.013_ = 0.028, *p* = 0.878) (Table S1). To test the effect of competition from competing mutations I measured the fitness of *spoT* ^Ev^ clones isolated from the two rec− lines at later time points, when their spread was much slower (Figure 2). Here, the relative fitness conferred by the *spoT* ^Ev^ alleles decreased significantly, from 3.4% to −2.6% in one line and from 4.6% to −0.1% in the other (Figure 3; Table 2). There was no corresponding change in fitness relative to the ancestor between these time points, so this decrease can only be explained by the spread of one or more beneficial mutations amongst the competing clones that did not have the focal mutation (*F*_1, 35_ = 1.007, *p* = 0.322) (Table S2). No significant difference was found when the fitness of *spoT* ^Ev^ clones isolated from rec+ lines at early and late time points was compared to contemporary clones that did not have the focal mutation (Table 3). Therefore, the effect of increased competition faced by focal beneficial mutations was specific to the absence of recombination.

**Table 2:**
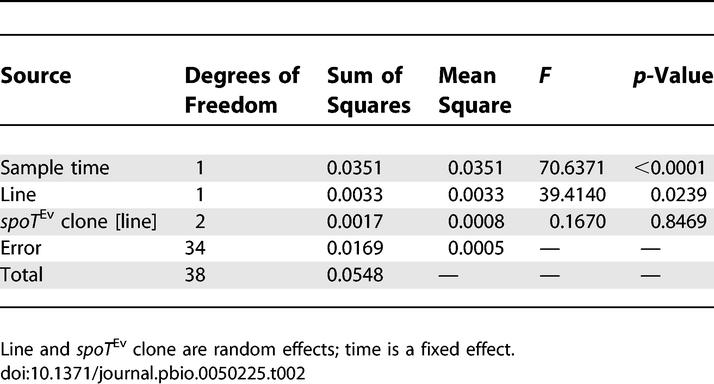
Comparison of Relative Fitness of *spoT* ^Ev^ Clones Isolated from Rec− Lines at Early and Late Sample Points

**Table 3:**
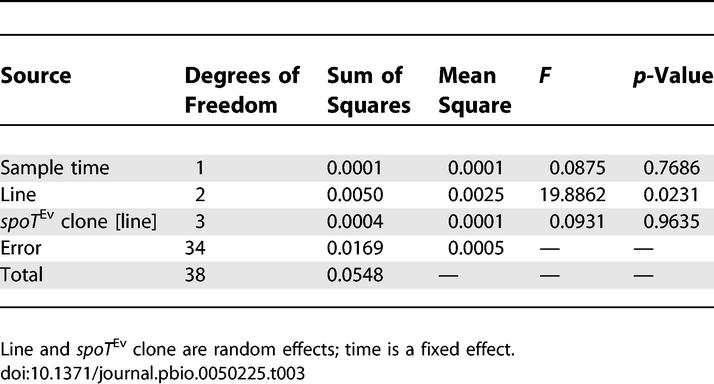
Comparison of Relative Fitness of *spoT* ^Ev^ Clones Isolated from Rec+ Lines at Early and Late Sample Points

The findings presented above indicate that multiple beneficial mutations were present in the evolving populations and that, as required by the FM model, in the absence of recombination, competition between these mutations was associated with slower fixation times. Indeed, this competition was so strong that in the rec− lines, the relative effect of the focal *spoT* ^Ev^ mutations became negative, such that additional beneficial mutations must have occurred on the same background in order for the focal mutation to fix. The possible existence of linked deleterious mutations cannot explain this result by itself, because the deleterious mutations would impose a constant cost to the focal mutation. By contrast, in the rec+ lines, the relative advantage conferred by the *spoT* ^Ev^ mutations showed no overall change, indicating that recombination effectively reduced the effect of competition between beneficial mutations. This reduction in competition is consistent with recombination bringing competing beneficial mutations together into one lineage, the mechanism proposed by the FM model.

Three additional lines of evidence support the interpretation that competition between beneficial mutations was a significant factor in the adaptation of the high mutation rate lines. First, there was a significant difference in relative fitness between the two clones containing a *spoT* ^Ev^ beneficial mutation in one rec− line, consistent with at least one additional beneficial mutation having arisen and reached an appreciable frequency within this lineage (*t*_8_ = −4.489, two-tailed *p* = 0.002) (sample 8 in Figure 3). Second, sequencing of *spoT* in the 16 low mutation rate lines found only one mutation. This frequency is marginally non-significantly lower than that of the five mutations found in the high mutation rate lines, consistent with there being a higher probability of fixing large effect mutations—which are generally expected to be less common—when beneficial mutations must compete for fixation [28] (Fisher's exact test, one-tailed *p* = 0.086). Third, the advantage conferred by *spoT* ^Ev^ alleles in the competitions carried out against contemporary clones is substantially lower than the advantage of ∼9.4% seen in competition with the ancestral strain (cf. [40]; Figure 3). This difference provides strong evidence that contemporary clones not having a focal mutation did contain alternative beneficial mutations. It is also worth noting that the relative fitness reductions seen in the rec− lines are substantially greater than the median fitness cost incurred by gene disruption mutations in the same strain (median effect of gene deletion, *s* = −0.014) [41].

It is important to note that the FM and mutation load models are not mutually exclusive. The results presented above support the FM model, but they do not rule out the possibility that removal of linked deleterious mutations may also have played a role in increasing the speed with which the focal beneficial mutation fixed in rec+ lines. One way to assess the potential importance of this process is to estimate the amount of within-population genetic variance in fitness, *w*_(gen)_, prevailing in the rec+ lines at the time when the focal mutations arose. For deleterious mutations to play an influential role in limiting the spread of these mutations, the benefit they confer must be small relative to this variance (*s* ≪ 6σ*w*_(gen)_ [35]). Removing linked deleterious mutations will only provide a substantial advantage if this condition is met. Using the fitness data reported above I estimated *w*_(gen)_ in the rec+ lines, based on those clones that did not have the focal *spoT* ^Ev^ mutation, when this mutation was at a low frequency. This measure is conservative because it combines differences between clones due to beneficial as well as deleterious mutations. I found that the genetic variance in fitness was clearly sufficient to influence the spread of the focal mutation in only one of the three rec+ populations (Table S3). Therefore, while deleterious mutations may have played some role in slowing adaptation in rec+ populations, they are not sufficient to explain the overall advantage to recombination.

The results reported in this study have important implications for bacterial adaptation. Competition between different lineages of the same species may be common in certain environments, for example, in clinical settings where adaptation to novel hosts can select for strains with high mutation rates [42,43]. In the absence of recombination, clonal interference means that increasing mutation rates will not generally cause a proportional increase in the rate of adaptation [16–18]. Conjugative plasmids are commonly found in clinical isolates and may reduce this interference by recombining competing beneficial mutations [44]. Plasmid transfer between lineages with different beneficial mutations may also contribute to continued selection for the plasmids themselves [45].

In summary, I found that recombination and mutation rate interact with each other in determining the speed of adaptive evolution. This finding supports the FM model for the evolution of sex, demonstrating that recombination increases the rate of adaptation only when competing beneficial mutations are present in the population. Also, I was able to identify a beneficial mutation that contributed to adaptation in a number of evolved populations. Comparing the dynamics of this mutation across recombination treatments allowed me to demonstrate directly that recombination reduced the amount of interference between a focal beneficial mutation and other competing beneficial mutations. This reduction in competition shortened the time needed for the focal mutation to fix in the population, providing an explanation for the observed benefit of recombination.

## Materials and Methods

### Plasmid and strain construction.

An F plasmid was obtained from the Coli Genetic Stock Center (Yale University, New Haven, Connecticut, United States), as an isolate from strain K603 (CGSC#6451). This plasmid, designated F1–10, encodes resistance to tetracycline and contains a portion of the E. coli genome encompassing the *lac* operon. This plasmid was chosen because this region of homology increases the rate at which the plasmid recombines into the host chromosome and therefore the frequency of chromosomal gene transfer. To make a rec− derivative of this plasmid, a non-polar *traD* deletion was introduced using a PCR-based approach [46]. This mutation decreased the frequency of plasmid transfer by more than 10^4^-fold and reduced the frequency of chromosomal gene transfer to undetectable levels [47]. This gene was chosen because its deletion does not affect plasmid pili production [48] and has been shown not to affect plasmid mutability [49]. The F plasmid imposes a fitness cost of ∼7% on the host cell during growth in minimal glucose medium [50]. To test if the *traD* deletion affected this cost, I performed a competition assay (see below) to measure the relative fitness of identical host cells carrying either the F plasmid or the F Δ*traD* derivative. This assay found that the cost of carriage was reduced slightly, but not significantly, by the *traD* mutation (difference in relative fitness of ancestral strain containing F − F Δ*traD* = −0.033, *t*_16_ = −1.430, one-tailed *p* = 0.086).

A previously described strain of E. coli B, REL606, was used as the host bacterium in the low mutation rate lines [51]. This strain carries no known plasmids or bacteriophage, and is therefore strictly asexual. Construction of a mutator derivative of this strain was carried out by P1 transduction of a disrupted allele of *mutS, mutS*::Tn*5,* into REL606 [52]. Disruption of the *mutS* allele does not have any measurable effect on fitness [53]. Rec+ and control rec− derivatives of REL606 and REL606 *mutS*::Tn*5* were made by using standard methods to separately introduce F and FΔ *traD* plasmids into both backgrounds to create the four ancestral strains used to found the evolution experiment. REL607, a spontaneous mutant of REL606 that is able to utilise arabinose, and a spontaneous nalidixic acid (Nx) resistant mutant of REL606 were obtained and used to allow selection of different strains in control assays designed to measure recombination rates (see below). Control experiments found that the rate of recombination was not affected by the *mutS*::Tn*5* allele.

### Culture conditions.

All incubations were carried out at 37 °C in 96 × 2-ml blocks (Qiagen; http://www.qiagen.com/) with shaking at 150 rpm. Each well was filled with 1 ml of medium. LB broth was used to grow cells from −80 °C freezer stocks. Davis minimal medium supplemented with glucose at 25 mg/l (DM25) was used at all other times.

### Recombination assays.

To measure the number of “gene equivalents” transferred between bacteria during each 1-d cycle of the evolution experiment, I introduced the F plasmid into two derivatives of REL606 that were distinguishable on the basis of unique markers (one strain was Nx resistant and unable to utilise the sugar arabinose [Ara–]; the other strain was sensitive to Nx but was able to utilise arabinose [Ara+]). These strains were inoculated from frozen stocks into LB medium and incubated for 24 h. Strains were then diluted 100-fold and 5 μl inoculated separately into DM25. Following a further 24 h of incubation, the two strains were mixed 1:1 and diluted 1:100 into fresh DM25 medium. After 24 h of co-incubation, cells were plated on minimal arabinose plates supplemented with Nx. Only cells that had recombined the Nx resistant and Ara+ markers could grow on this plate. To estimate the total amount of horizontal gene transfer, the number of recombinant cells was first multiplied by the number of genes separating the two markers. Assuming that only half of these transmitted genes become incorporated into the recipient cell's chromosome [54] and that genes outside these markers were not transferred, this number was divided by two to arrive at a conservative estimate for the total number of genes incorporated into the recipient cell chromosome. This calculation gives an estimate of chromosomal gene transfer between rec+ cells of ∼1 × 10^−4^ genes/cell/generation in the evolution environment. Although this rate might seem low, it is nevertheless several orders of magnitude higher than the spontaneous mutation rate, even in the presence of the *mutS* mutation [18,38]. It is important to note that this assay was performed between donor and recipient cells that both contained the F plasmid; therefore, this estimate of gene transfer includes any effect of plasmid surface exclusion in reducing the frequency of conjugation between plasmid-containing cells.

### Evolution experiment.

The evolution experiment consisted of eight replicate lines started with each of four ancestral strains: REL606 (F) (low mutation rate, rec+), REL606 (F Δ*traD*) (low mutation rate, rec−), REL606 *mutS*::Tn*5* (F) (high mutation rate, rec+), and REL606 *mutS*::Tn*5* (F Δ*traD*) (high mutation rate, rec−). All lines were propagated in 1 ml of DM25 medium in 96 × 2-ml blocks. Each day 5 μl of culture was transferred to 1 ml of fresh medium, allowing ∼7.64 generations per day (= log_2_ 200). This environment and strain combination corresponds to a relative mutation supply (calculated as the product of effective population size and mutation rate) of ∼2.9 in the low mutation rate lines and ∼101.1 in the high mutation rate lines with respect to Figure 2 of de Visser et al. [18]. Propagation was continued for 130 d, to give a total of ∼1,000 generations of evolution. Every 13 d (∼100 generations), following transfer to a fresh block, glycerol was added to all lines, which were then stored at −80 °C. All lines were initially genetically uniform; therefore, all adaptation arose through de novo mutation.

Several precautions were taken to eliminate the possibility of external contamination or cross-contamination during the evolution experiment. The ancestral strain contained several characteristic genetic markers that were checked every ∼100 generations throughout the experiment [51]. At no time were any bacteria observed to differ from the ancestral marker profile; therefore, it is very unlikely that there was any external contamination. I also checked for the continued presence of F and F Δ*traD* plasmids by screening evolved bacteria for the plasmid-encoded tetracycline resistance marker. In no case did the frequency of plasmid carriage drop below ∼90%. Monitoring for cross-contamination between lines was facilitated by the arrangement of the evolving populations in a checker-board pattern in the propagation block. In this arrangement the four wells nearest to each population contained uninoculated medium. Observation of bacterial growth in these wells provided a sensitive means by which to observe any splash that could potentially contaminate adjacent wells. On several occasions such contamination was observed; in these cases the experiment was restarted from the previous day's block, which had been kept overnight at 4 °C. As an additional precaution, mutation rate lines that differed by the presence or absence of the kanamycin resistance marker were grown adjacent to one another. Lines were periodically plated to kanamycin-supplemented medium to detect cross-contamination.

Following 1,000 generations of evolution, several assays were carried out to determine if mutation and recombination rates remained at ancestral values. Recombination rates were assayed as described above, except evolved strains were co-incubated with a reference strain that carried a non-synonymous mutation in the gene *galK,* rendering them unable to use galactose as a sole carbon source. Recombinants were selected on Davis minimal medium supplemented with Nx and galactose. Mutation rates were assayed as described previously [18]. Mutation rates to Nx resistance and arabinose utilisation were calculated and averaged to estimate the overall mutation rate. No significant changes in either recombination or mutation rates were observed in any of the evolved lines.

### Fitness assays.

The fitness of evolved strains relative to the ancestor was assayed by competitions carried out in the same conditions prevailing during the evolution experiment. All evolved strains were Ara−, to allow these strains to be distinguished from their ancestors; derivatives of the ancestral strains were selected that were Ara+. These two marker types can be distinguished by plating on tetrazolium arabinose indicator medium. On this medium, cells that can utilise arabinose form white colonies, whereas cells that cannot use arabinose form red colonies. The arabinose marker is selectively neutral in the evolution environment [51]. Before each fitness assay, the two competitors were acclimated to the competition environment by growing them separately under the same environmental conditions to be used in the competition. Competitors were then mixed by diluting 400-fold into fresh DM25 medium and a sample immediately plated on tetrazolium arabinose agar to estimate the initial densities of the competing strains. At the end of 1 d of competition (i.e., one propagation cycle), a further sample was plated on tetrazolium arabinose agar to obtain the final density of each competitor. The fitness of the evolved strain relative to the ancestor was calculated as ln(*N*_E_(1)/*N*_E_(0))/ln(*N*_A_(1)/*N*_A_(0)), where *N*_E_(0) and *N*_A_(0) represent the initial densities of the evolved and ancestral strains, respectively, and *N*_E_(1) and *N*_A_(1) represent corresponding densities at the end of the competition. All competitions between ancestral and evolved clones were carried out with 10-fold replication.

Competitions involving evolved clones that did and did not have the *spoT* ^Ev^ allele were performed similarly. All clones were initially Ara−. To allow the two types to be distinguished from one another, spontaneous Ara+ revertants were selected from those clones that had the *spoT* ^Ev^ allele and used in competitions. These competitions were carried out over two or four transfer cycles to increase the precision of fitness estimates. Competitions were carried out with 3-fold replication.

### Sequencing and mutation dynamics.

Primers were designed to amplify overlapping fragments of the gene *spoT* including upstream regulatory regions. Purified PCR products were sequenced on an Applied Biosystems (http://www.appliedbiosystems.com/) 3130XL capillary sequencer. Three clones were assayed at the final time point in each high mutation rate line. I sequenced *spoT* in three randomly chosen clones isolated from each of the 32 lines in the high and low mutation rate treatments and found mutations in four rec+ and two rec− lines in the high mutation rate treatment and one rec− line in the low mutation rate treatment. In one high mutation rate treatment rec− line, the *spoT* ^Ev^ allele had not fixed by 1,000 generations. This line was continued for a further 300 generations, after which time the line was screened again and the allele was found to have fixed. The mutations found all caused amino acid substitutions in a region of SpoT involved in the synthesis of the “alarmone” molecule (p)ppGpp [55], but no two lines shared the same mutation. Dynamics of mutations were tracked using a combination of RFLP- and PCR-based approaches on clones isolated from those lines in which mutations were found. For mutations that changed a restriction site I amplified a region around this site, digested this fragment, and assayed for ancestral or evolved restriction patterns. In cases where no restriction site was introduced I developed PCR approaches taking advantage of the reduced binding and extension efficiency that occurs if there is a mismatch at the 3′ end of a PCR primer. All assays were performed at least twice for each clone, and positive and negative controls were included in every block. At least 100 clones were screened at the time point immediately preceding the first detected occurrence of a mutation and at the point at which the mutation had apparently fixed. Absence of mutant and progenitor types in this sample size indicates they are unlikely to be present in the population at greater than 3.1% (Wald test, 95% confidence interval 0%–3.1%). At least 45 clones were screened at each intermediate time point.

### Statistical methods.

Mixed models were run to test the interaction between recombination and mutation rate, with replicate evolved line nested within recombination and mutation rate treatments. Mixed models were also used to test the null hypotheses that (i) there was no effect of recombination treatment on the relative fitness of clones having a *spoT* ^Ev^ allele isolated from early time points and (ii) there was no effect of sample time (early versus late) on the relative fitness of clones having a *spoT* ^Ev^ allele in the absence of recombination. In all cases denominator degrees of freedom was estimated using a Satterthwaite approximation.

## Supporting Information

Table S1 Comparison of Contemporary Fitness of *spoT* ^Ev^ Clones Isolated from Rec+ and Rec− Lines at an Early Sample Point (30 KB DOC)

Table S2 Comparison of Relative Fitness of *spoT* ^Ev^ Clones Isolated from Rec− Lines at Early and Late Time Points (30 KB DOC)

Table S3 Within-Population Genetic Variance in Fitness among Clones Not Having the *spoT* ^Ev^ Mutation in Rec+ Lines (29 KB DOC)

## Abbreviations

Ara+: able to utilise arabinose
Ara−: unable to utilise the sugar arabinose
FM: Fisher–Muller
Nx: nalidixic acid
rec+: recombination proficient
rec−: recombination deficient
*spoT*^Ev^: evolved *spoT*
