# Supplementary material for: Recombination Speeds Adaptation by Reducing Competition between Beneficial Mutations in Populations of Escherichia coli
Source: PLoS Biol. 2007 Aug 21;5(9):e225. doi: 10.1371/journal.pbio.0050225 (PMC1950772; doi:10.1371/journal.pbio.0050225)
Supplement: Supplementary file 1 [file pbio.0050225.st001.doc]

**Table S1.** **Comparison of fitness, measured relative to competing clones, of *spoT^Ev^* clones isolated from rec+ and rec- lines at an early sample point.** Line and *spoT^Ev^* clone are random effects, recombination is a fixed effect.

| Source | df | SS | MS | F | P |
| --- | --- | --- | --- | --- | --- |
| Recombination | 1 | 0.0002 | 0.0002 | 0.0280 | 0.8777 |
| Line[recombination] | 3 | 0.0185 | 0.0062 | 80.4968 | 0.0002 |
| *spoT^Ev^* clone[recombination,line] | 5 | 0.0004 | 0.0001 | 0.0993 | 0.9917 |
| Error | 39 | 0.0308 | 0.0008 |  |  |
| Total | 48 | 0.0498 |  |  |  |
