# Supplementary material for: Recombination Speeds Adaptation by Reducing Competition between Beneficial Mutations in Populations of Escherichia coli
Source: PLoS Biol. 2007 Aug 21;5(9):e225. doi: 10.1371/journal.pbio.0050225 (PMC1950772; doi:10.1371/journal.pbio.0050225)
Supplement: Supplementary file 2 [file pbio.0050225.st002.doc]

**Table S2. Comparison of fitness, measured relative to the ancestor, of *spoT^Ev^* clones isolated from rec- lines at early and late time points relative to their ancestor.** Line and *spoT^Ev^* clone are random effects, time is a fixed effect.

| Source | df | SS | MS | F | P |
| --- | --- | --- | --- | --- | --- |
| Sample time | 1 | 0.0014 | 0.0014 | 1.0077 | 0.3223 |
| Line | 1 | 0.0032 | 0.0032 | 3.5602 | 0.1998 |
| *spoT^Ev^* clone[line] | 2 | 0.0018 | 0.0009 | 0.6531 | 0.5266 |
| Error | 35 | 0.0476 | 0.0014 |  |  |
| Total | 39 | 0.0539 |  |  |  |
