# Supplementary material for: Recombination Speeds Adaptation by Reducing Competition between Beneficial Mutations in Populations of Escherichia coli
Source: PLoS Biol. 2007 Aug 21;5(9):e225. doi: 10.1371/journal.pbio.0050225 (PMC1950772; doi:10.1371/journal.pbio.0050225)
Supplement: Supplementary file 3 [file pbio.0050225.st003.doc]

**Table S3. Genetic variance in fitness among clones not having the *spoT^Ev^* mutation in rec+ lines.** Variance components (w_(gen)_) were calculated using a maximum likelihood approach.

| Time point (from Figure 2) | w_(gen)_ (x 10^4^) | 6**σ_W(gen)_** |
| --- | --- | --- |
| 1 | 19.20 | 0.263 |
| 3 | 4.320 | 0.124 |
| 5 | -0.035^*^ | - |

*Negative variance component represents statistical noise and is not biologically meaningful.
